# Structural, Electronic, and Optical Properties of BiOX_1−*x*_Y_*x*_ (X, Y = F, Cl, Br, and I) Solid Solutions from DFT Calculations

**DOI:** 10.1038/srep31449

**Published:** 2016-08-23

**Authors:** Zong-Yan Zhao, Qing-Lu Liu, Wen-Wu Dai

**Affiliations:** 1 Faculty of Materials Science and Engineering, Kunming University of Science and Technology, Kunming 650093, People’s Republic of China; 2 Yunnan Key Laboratory of Micro/Nano Materials & Technology, School of Materials Science and Engineering, Yunnan University, Kunming 650504, People’s Republic of China; 3 Key Laboratory of Nanodevices and Applications, Suzhou Institute of Nano-Tech and Nano-Bionics, Chinese Academy of Sciences, Suzhou 215123, People’s Republic of China

## Abstract

Six BiOX_1−*x*_Y_*x*_ (X, Y = F, Cl, Br, and I) solid solutions have been systematically investigated by density functional theory calculations. BiOCl_1−*x*_Br_*x*_, BiOBr_1−*x*_I_*x*_, and BiOCl_1−*x*_I_*x*_ solid solutions have very small bowing parameters; as such, some of their properties increase almost linearly with increasing *x*. For BiOF_1−*x*_Y_*x*_ solid solutions, the bowing parameters are very large and it is extremely difficult to fit the related calculated data by a single equation. Consequently, BiOX_1−*x*_Y_*x*_ (X, Y = Cl, Br, and I) solid solutions are highly miscible, while BiOF_1−*x*_Y_*x*_ (Y = Cl, Br, and I) solid solutions are partially miscible. In other words, BiOF_1−*x*_Y_*x*_ solid solutions have miscibility gaps or high miscibility temperature, resulting in phase separation and F/Y inhomogeneity. Comparison and analysis of the calculated results and the related physical–chemical properties with different halogen compositions indicates that the parameters of BiOX_1−*x*_Y_*x*_ solid solutions are determined by the differences of the physical–chemical properties of the two halogen compositions. In this way, the large deviation of some BiOX_1−*x*_Y_*x*_ solid solutions from Vegard’s law observed in experiments can be explained. Moreover, the composition ratio of BiOX_1−*x*_Y_*x*_ solid solutions can be measured or monitored using optical measurements.

Energy shortages and environmental pollution have resulted in the development and utilization of new energy sources. In particular, photocatalytic technology has attracted considerable interest. Under solar light irradiation, photocatalysts can decompose water to produce hydrogen, convert greenhouse gas (i.e., carbon dioxide) into hydrocarbon fuel, and decompose harmful gases and pollutants in water to harmless inorganic substances. That is, photocatalysis can use solar energy to alleviate energy shortage and environmental pollution. Although this is an attractive prospect, the current development of photocatalytic technology faces two major bottlenecks. First, most of current conventional photocatalysts are wide band-gap semiconductor materials. They only exhibit photocatalytic activity under UV-light irradiation, meaning they cannot fully utilize solar energy. Second, because of the existence of impurities and defects, the recombination rate of the photogenerated electron–hole pairs is high, leading to very low quantum efficiency of solar energy conversion. To realize the efficient use of solar energy, either conventional photocatalysts need to be modified, or novel photocatalysts need to be developed.

Bismuth oxyhalides (BiOX, X = F, Cl, Br, and I) are layered semiconductors. The structure of BiOX compounds is composed of strong intralayer interactions within [Bi_2_O_2_]^2+^ slabs and weak van der Waals interlayer interactions between adjacent X^−^ slabs. The interleaved [Bi_2_O_2_]^2+^ and X^−^ slabs along the *c*-axis direction produce an internal electric field, which remarkably accelerates the transfer and reduces the recombination rate of photogenerated electron–hole pairs. In addition, they do not contain toxic and heavy metals. Therefore, they have numerous applications, such as in pigments in the cosmetic industry, magnetic materials, pharmaceuticals, and catalysts in the oxidative coupling of methane reaction^1^,^2^,^3^,^4^,^5^. Because of the above advantages, BiOX compounds have attracted much attention in the field of photocatalysis^6^,^7^,^8^. In 2008, Zhang *et al*. found that all of the BiOX (X = Cl, Br, and I) compounds exhibit photocatalytic activity and BiOI exhibits excellent activity under both UV–vis and visible-light irradiation^3^. The preparation, properties, and photocatalytic performance of BiOX compounds have subsequently been extensively investigated^9^,^10^,^11^,^12^,^13^,^14^,^15^,^16^.

BiOX compounds have the same structure and similar characteristics. The band gaps of BiOX compounds decrease from ~3.9 to ~1.8 eV with increasing atomic number of the halogen^17^. This unique feature provides the opportunity to tailor the basic photoelectric physical properties and photocatalytic performance of BiOX compounds by intentionally mixing halogens. In other words, BiOX_1−*x*_Y_*x*_ (X, Y = F, Cl, Br, and I) solid solutions (or semiconductor alloys) may meet the requirements for photocatalytic applications. Semiconductor solid solutions have some benefits over other band-gap tailoring techniques (e.g., impurity doping) because it is possible to alter the electronic properties without introducing impurity states that could act as recombination centres. Moreover, semiconductor solid solutions provide a natural way of tuning the magnitude of the band-gap energy and other photoelectric properties to optimize and increase the application of semiconductor devices. The most successful example in the field of photocatalysis are (Ga_1−*x*_Zn_*x*_)(N_1−*x*_O_*x*_) solid solutions, which show excellent photocatalytic performance for hydrogen production from photocatalytic water splitting under visible-light irradiation, while the two components (GaN and ZnO) only absorb UV-light^18^,^19^. Generally, choosing a solid-solution partner can be difficult because elements with partially filled valence shells that can accept electrons or holes risk reducing the overall photocurrent for redox reactions. However, the features of BiOX compounds can avoid these problems. Recently, it has been reported that BiOX compounds are able to form solid solutions through various synthesis processes^20^,^21^,^22^,^23^,^24^,^25^,^26^,^27^,^28^,^29^,^30^,^31^,^32^,^33^,^34^. For example, Keller *et al*. found that there are no quaternary phases in BiOX_1−*x*_Y_*x*_ (X, Y = Cl, Br, and I) solid solutions, BiOCl_1−*x*_Br_*x*_ and BiOBr_1−*x*_I_*x*_ form systems of unlimited mutual solubility, and BiOCl_1−*x*_I_*x*_ has limited solubility when it is iodine rich^20^. Ren *et al*. demonstrated that optimized BiOX_1−*x*_Y_*x*_ solid solutions possess higher photocatalytic activity than pure BiOX compounds because of the wider range of the visible light response and the reduced recombination rate of electron–hole pairs^31^.

Although the synthesis and photocatalytic performance of BiOX_1−*x*_Y_*x*_ solid solutions have been investigated by independent research groups, the basic concepts and determination of the photocatalytic performance have been hampered by a lack of fundamental knowledge about BiOX_1−*x*_Y_*x*_ solid solutions. Furthermore, studies have been limited to one or two types of BiOX_1−*x*_Y_*x*_ solid solution. To understand the structural and electronic properties of BiOX_1−*x*_Y_*x*_ solid solutions, it is necessary to systematically investigate their properties using theoretical calculations or simulations. However, only a few theoretical articles have been reported. Zhang *et al*. calculated the electronic structures of BiOX_1−*x*_Y_*x*_ solid solutions by density functional theory (DFT), and considered that the alloying effect in BiOX results in a substantially lower electron–hole recombination rate and much higher photocatalytic efficiency^35^. Their work is of great practical value, and provides a reasonable explanation for some experimental observations. However, they did not provide a basic rule for BiOX_1−*x*_Y_*x*_ solid solutions. In particular, they did not analyse the large deviation of some BiOX_1−*x*_Y_*x*_ solid solutions from Vegard’s rule reported by Keller *et al*.^20^.

The main purpose of this study is to provide a comprehensive understanding of BiOX_1−*x*_Y_*x*_ solid solutions for photocatalysis applications. The topics discussions in this article include the structural, electronic, and optical properties of BiOX_1−*x*_Y_*x*_ solid solutions, and their relationship with photocatalytic performance. We also provide a possible explanation for previous experimental observations and some useful data for tailoring the properties of BiOX photocatalysts.

## Results and Discussions

### Formation energy

**Figure 1:**
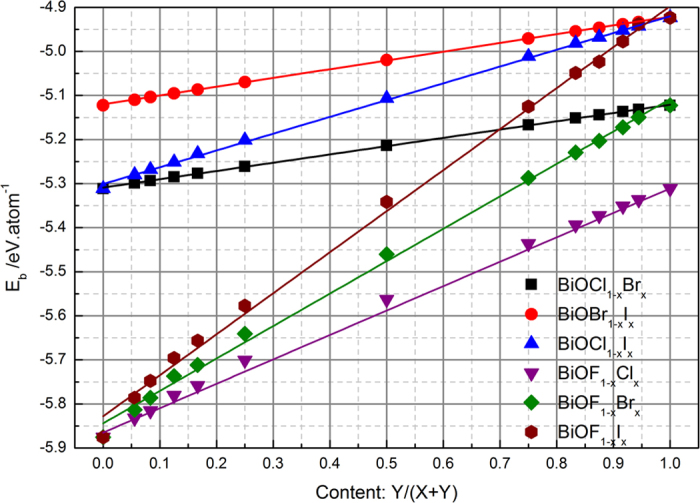
Binding energy of BiOX_1−__*x*_Y_*x*_ solid solutions as a function of content *x*.

Isolated atoms can combine to form a crystal because the combined system has lower energy. That is, the free atoms combined in a crystal will release energy, or decomposition of a crystal requires energy. This energy is referred to the binding energy (*E*_b_) defined as following for BiOX_1−*x*_Y_*x*_ solid solutions:


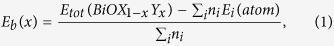


where *E*_tot_(BiOX_1−*x*_Y_*x*_) is the total energy of the BiOX_1−*x*_Y_*x*_ solid solution. If *x* = 0, it is the total energy of pure BiOX. *n*_*i*_ and *E*_*i*_(atom) are the number and energy of an isolated *i* atom, respectively. The definition of *E*_b_ indicates that the crystal structure of a solid solution is more stable when the value of the binding energy is larger. The calculated binding energies of the BiOX_1−*x*_Y_*x*_ solid solutions are shown in Fig. 1. In the cases of the pure BiOX compounds, BiOF has the largest binding energy. The binding energy decreases with increasing atomic number of the halogen. This may be related to fluorine having the highest electronegativity, resulting in the van der Waals interactions between the [Bi_2_O_2_]^2+^ slabs of BiOF are the largest. So, the lattice constant of BiOF along the *c* axis is the smallest. The van der Waals interactions between [Bi_2_O_2_]^2+^ slabs of BiOI are the smallest, and its lattice constant along the *c* axis is the largest. For BiOX_1−*x*_Y_*x*_ solid solutions, when Y is incorporated into BiOX, the value of *E*_b_ increases, indicating that the incorporation of heavy halogen atoms will decrease the stability of the crystal. With increasing Y content, the binding energies of all of the BiOX_1−*x*_Y_*x*_ solid solutions linearly increases. In the complete range of the Y content, the variation of the binding energy of the BiOCl_1−*x*_Br_*x*_ solid solutions is the smallest (~0.19 eV/atom), while that of the BiOF_1−*x*_I_*x*_ solid solutions is the largest (~0.95 eV/atom). These calculated results indicate that only small energy is needed to incorporate Y into the BiOX (X = Cl and Br) crystal matrix to form solid solution. The above phenomena are quantitatively reflected by the fitting curves in Fig. 1 and the fitting parameters in Table 1: the correlation coefficients for all of the linear fitting curves are greater than 0.99 and the slope of the curve is smallest for the BiOCl_1−*x*_Br_*x*_ solid solutions and largest for the BiOF_1−*x*_I_*x*_ solid solutions.

**Figure 2:**
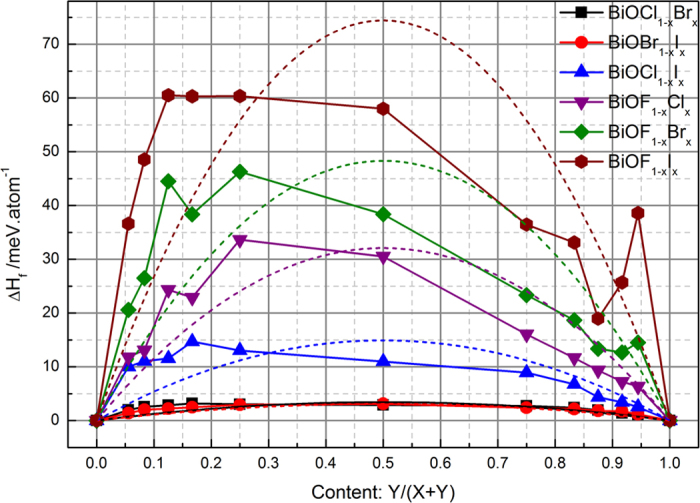
Formation enthalpy of BiOX_1−__*x*_Y_*x*_ solid solutions as function of content content *x*.

To describe the miscibility of the BiOX_1−*x*_Y_*x*_ solid solutions, the formation enthalpy was also calculated:


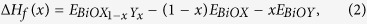


where *E*_BiOX_ and *E*_BiOY_ are the total energies of pure BiOX and BiOY with the same size of supercell, and *E*_BiOX_1−*x*_Y_*x*__ is the total energy of the BiOX_1−*x*_Y_*x*_ solid. The calculated results are shown in Fig. 2. The formation enthalpy Δ*H*_f_(*x*) describes the energy cost of mixing X and Y halogens in a certain lattice. It is clear that all of the BiOX_1−*x*_Y_*x*_ solid solutions have an upward bowing in their Δ*H*_f_ dependence on *x*, which indicates they prefer decoherent phase separation into BiOX and BiOY at zero temperature. Comparing these calculated curves, the formation enthalpies of the BiOX_1−*x*_Y_*x*_ solid solutions are in the order BiOCl_1−*x*_Br_*x*_ < BiOBr_1−*x*_I_*x*_ < BiOCl_1−*x*_I_*x*_ < BiOF_1−*x*_Cl_*x*_ < BiOF_1−*x*_Br_*x*_ < BiOF_1−*x*_I_*x*_ for the same *x*. This indicates that halogen mixing is easier with halogens with similar size. It is worth noticing that there is only one peak for BiOCl_1−*x*_Br_*x*_, BiOBr_1−*x*_I_*x*_, and BiOCl_1−*x*_I_*x*_, while there is more than one peak for the other three types of BiOF_1−*x*_Y_*x*_ solid solution. These peaks are located on the light-halogen-rich side of the Δ*H*_f_ against the *x* curve (*x* < 0.5), indicating that limited solubility might occur on the heavy-halogen-rich side (*x* > 0.5). According to conventional solid-solution theory, the solid-solution formation enthalpy is almost a quadratic function of *x*:^36^


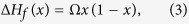


where Ω is the interaction parameter, which is an indicator of the solid-solution solubility. A larger Ω indicates a smaller solubility. The fitting curves are shown in Fig. 2 as dashed lines, and the corresponding fitting parameters are listed in Table 1. The fitting curves for BiOCl_1−*x*_Br_*x*_ and BiOBr_1−*x*_I_*x*_ solid solutions have very large correlation coefficients, while the other four BiOX_1−*x*_Y_*x*_ solid solutions have small correlation coefficients. The Ω values of BiOCl_1−*x*_Br_*x*_ and BiOBr_1−*x*_I_*x*_ are very small and almost the same, suggesting that component-uniform BiOCl_1−*x*_Br_*x*_ and BiOBr_1−*x*_I_*x*_ solid solutions can be easily prepared at the standard growth temperature. The Ω values of the other four solid solutions are relatively large. Furthermore, according to the standard solid-solution model, the miscibility gap temperature is given by Ω (per mixed atom)/2*k*_B_*T*^37^. Using this equation, the transition temperature is about 150 K for BiOCl_1−*x*_Br_*x*_ and BiOBr_1−*x*_I_*x*_ solid solutions, and 700 K for BiOCl_1−*x*_I_*x*_ solid solutions. These miscibility gap temperatures are less than or close to the typical growth temperatures in the common preparation process, suggesting that these three types of solid solution can be easily prepared. However, for the other three types of solid solution containing fluorine, the transition temperatures are above 1400 K, suggesting that BiOF_1−*x*_Y_*x*_ solid solutions are difficult to prepare in practice and have relatively large miscibility gaps. This may be the reason why there is still no relevant experimental work in the literature. In other words, good miscibility is expected for BiOCl_1−*x*_Br_*x*_ and BiOBr_1−*x*_I_*x*_ solid solutions, and thus it is expected that homogeneous unlimited solid solutions can be formed. For BiOCl_1−*x*_I_*x*_ solid solutions, component-uniform samples with variable compositions can be synthesized, but homogeneous limited solid solutions can only be formed for certain compositions. The above calculated results are in good agreement with experimental observations^20^. Owing to the relatively large Ω values (i.e., miscibility gap temperatures) and multiple peaks in the Δ*H*_f_(*x*) curve, phase separation and component inhomogeneity are a common problem in the production of BiOF_1−*x*_Y_*x*_ solid solutions.

### Structural properties

A solid solution with two components can be called a binary alloy or a quasibinary alloy. The resulting solid solution generally has significantly different properties from those of its components. DFT calculations allow an expression for the variation of the crystal lattice constants of BiOX_1−*x*_Y_*x*_ solid solutions in the complete range of *x* to be constructed. For the pure BiOX compounds, the calculated lattice parameters are in good agreement with experimental measurements^17^. We compare the calculated results and the available experimental results^20^,^22^,^25^,^31^ in Figures S1 to S6 (in the Supporting Information). The calculated results are consistent with the experimental results, especially the variation trend with *x*, indicating that the supercell models chose in the present work are basically reasonable. For the DFT calculations, the size of supercell and the occupying sites of Y in the supercell also impact the final total energy of models and the lattice parameters. So, it should carefully choose the configuration of solid solution structure. In the present work, we found that if the model has higher symmetry, the total energy per cell is smaller. In this configuration, the Y atoms gather together at the same plane as much as possible. Thus, it is could be assumed the interaction between same halogen atoms (X-X or Y-Y) or different halogen atoms (X-Y) lead the variation of total energy per cell, as well as the variation of lattice constants. However, the interaction between halogen atoms are very small as mention in ref. 20., so the differences between the possible supercells for the same composition are relatively small (<0.5 eV/cell, one cell contains 6 atoms). Of course, there are some differences between experimental measurements and DFT calculation, which can be ascribed to the following two aspects: (1) the uncertainty of the method of experimental measurement. The XRD characterization is the most conventional method in experimental measurement, which is a statistical method of analysis, and the accuracy of the results depends on the degree of proficiency of researcher. Fox example, in the Figure S6, the lattice parameter of BiOCl_1−x_I_x_ solid solution has two different values at the x = 0.4. (2) The choice of supercell model (including size and occupation pattern) and ordered solid solution have limitation, impacting the accuracy of DFT calculations. For example, the choice of models is not equal interval based on the value of x, so the continuity of variation trend cannot be determined in the present work. However, from the general situation, the variation trends of experimental measurements and DFT calculations are still basically consistent with each other. Thus, the calculated results in the present work can partly explain some experimental phenomena observed previously.

**Figure 3:**
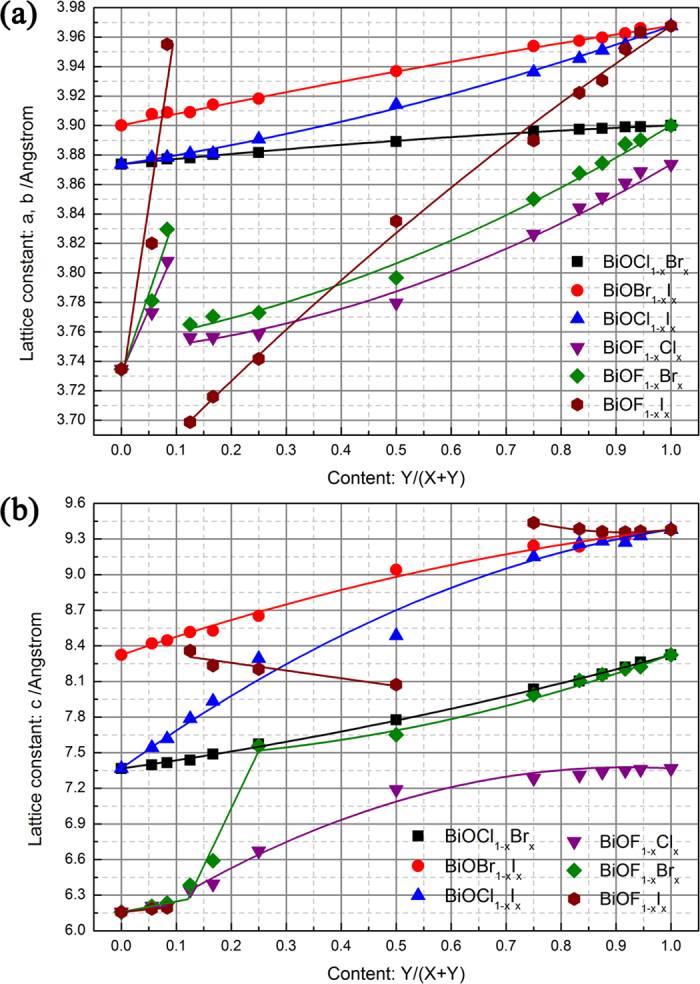
Lattice constants of BiOX_1−__*x*_Y*_x_* solid solutions as function of content *x*.

Figure 3 shows how the lattice constants vary with *x*. In the present work, the relationship between the lattice constants and the composition do not follow a linear relationship (i.e., a first-order function^20^). Therefore, the second-order function of Vegard’s law was used to fit these data:^38^


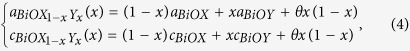


where *θ* is the bowing parameter. The fitting curves are plotted in Fig. 3, and the detailed fitting parameters are listed in Table 2. For BiOCl_1−*x*_Br_*x*_ and BiOBr_1−*x*_I_*x*_ solid solutions, the bowing parameters are very small. That is, the lattice constants almost linearly increase with increasing *x* and almost obey the first-order function of Vegard’s law. For the BiOCl_1−*x*_I_*x*_ solid solutions, the bowing parameter is larger than those of the former two cases. The variations of the lattice constants for BiOF_1−*x*_Y_*x*_ solid solutions are complicated. In the range 0 ≤ *x* ≤ 1/12, all of the lattice constants linearly increase with increasing *x*. Moreover, the slopes also increase with increasing atomic number of Y, except for lattice constant *c* of the BiOF_1−*x*_I_*x*_ solid solution. In the range 1/8 ≤ *x* ≤ 1, the variations of lattice constant *a* of all three BiOF_1−*x*_Y_*x*_ solid solutions and lattice constant *c* of the BiOF_1−*x*_Cl_*x*_ solid solutions obey the second-order function of Vegard’s law. Lattice constant *c* of the BiOF_1−*x*_Br_*x*_ solid solutions increases with increasing *x* in the range 1/8 ≤ *x* ≤ 1/4, while lattice constant *c* of the BiOF_1−*x*_I_*x*_ solid solutions slowly decreases with increasing *x* in the range 1/8 ≤ *x* ≤ 1/2. In the rest of the range of *x*, lattice constant *c* of the BiOF_1−*x*_Br_*x*_ and BiOF_1−*x*_I_*x*_ solid solutions obey the second-order function of Vegard’s law with a relatively large bowing parameter. It should be noted that for the BiOX_1−*x*_Y_*x*_ (X, Y = Cl, Br, and I) solid solutions, the bowing parameters of lattice constant *a* are smaller than those of lattice constant *c*. The situation for the BiOF_1−*x*_Y_*x*_ (Y = Cl, Br, and I) solid solutions is completely different. In the first linear variation region, the slopes of lattice constant *a* are larger than those of lattice constant *c*, especially in the case of the BiOF_1−*x*_I_*x*_ solid solutions. In contrast, the bowing parameters of lattice constant *a* are smaller than those of lattice constant *c* in the parabolic variation region.

**Figure 4:**
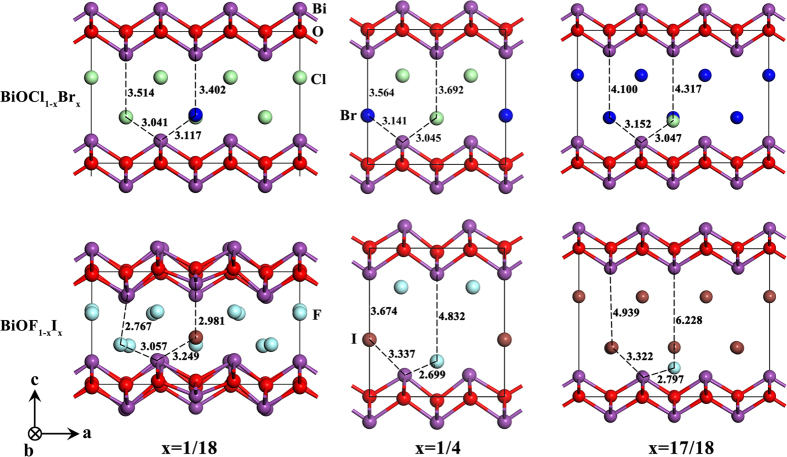
Crystal structures of BiOCl_1−__*x*_Br_*x*_ and BiOF_1−__*x*_I_*x*_ with *x* = 1/18, 1/4, and 17/18.

In the above relationship between formation energy and *x*, the lattice mismatch between the two components of the BiOX_1−*x*_Y_*x*_ solid solution is not the deciding factor for the variations of the lattice constants. For example, the *c* lattice mismatch between BiOCl and BiOI is 21.46% and the variations of the lattice constants of the BiOCl_1−*x*_I_*x*_ solid solution obey the second-order function of Vegard’s law in the complete range of *x*. However, although the *c* lattice mismatch between BiOF and BiOCl is 16.42%, the variations of the lattice constants of the BiOF_*1−x*_Cl_*x*_ solid solutions follow different rules. Keller *et al*. observed a large deviation from Vegard’s rule for the *c* lattice constant in BiOCl_1−*x*_I_*x*_ solid solutions and assumed that the strong bowing of *c*(*x*) is mainly because of the weak anion–anion interactions across the interface between two vicinal sandwiches^20^. Their assumption is mainly based on the one-sidedness of the halogen coordination polyhedral induced by weak van der Waals attractions between halogen atoms, which is hindered by strong X–X and X–O repulsions and by the rigidity of the [Bi_2_O_2_]^2+^ layers. The crystal structures of two typical examples are shown in Fig. 4. The bowing parameter is the smallest for the BiOCl_1−*x*_Br_*x*_ solid solutions, while the deviation from Vegard’s rule is the largest for the BiOF_1−*x*_I_*x*_ solid solutions. For the BiOCl_1−*x*_Br_*x*_ solid solutions, the Cl–Bi_1_ (~3.041–3.047 Å) and Br–Bi_1_ (~3.117–3.152 Å) bond lengths are almost constant with increasing *x*, while the Cl–Bi_2_ (~3.514–4.317 Å) and Br–Bi_2_ (~3.402–4.100 Å) bond lengths linearly increase with increase *x*. Furthermore, in the complete range of *x*, the distance between Cl and Br along the *c* axis is very small (~0.218 Å) and the [Bi_2_O_2_]^2+^ layers are hardly affected (no obvious distortion). In contrast to the above situation, for the BiOF_1−*x*_I_*x*_ solid solutions, the F–Bi_1_ (~2.699–3.057 Å), I–Bi_1_ (~3.249–3.337 Å), F–Bi_2_ (~2.767–6.288 Å), and I–Bi_2_ (~2.981–4.939 Å) bond lengths are in a relative large range, and these variations do not follow a definite trend with increasing *x*. Furthermore, the distance between F and I along the *c* axis varies from 0.393 to 1.285 Å, and the [Bi_2_O_2_]^2+^ layers are greatly affected (obvious distortion) for the F-rich compositions (*x* = 1/18 and 1/4). The microstructure variation of the other BiOX_1−*x*_Y_*x*_ solid solutions are within the above two extremes. These calculated results indicate that the macroscopic variations of the lattice constants of BiOX_1−*x*_Y_*x*_ solid solutions are determined by changes of the internal microstructure, and confirm the assumptions of Keller *et al*.

In the previous experimental report ref. 20, the authors found the BiOCl_1−x_I_x_ solid solutions also show abrupt changes for the cell parameters, and they assumed that the strong bowing or deviation of c(x) is mainly due to the weakness of anion-anion interaction across the interface between two vicinal sandwiches. In our present work, we have strengthened this view, and found that the interactions between the halogen atoms, as well as the interaction between the halogen atom and [Bi_2_O_2_]^2+^ layers, are closely related to the nature of the halogen atom itself. So we list these parameters and their differences in Table 3. Furthermore, at the different solubility or content of Y/(X + Y), these differences have different influence on the behavior of solid solutions as shown in Fig. 4. Comparison of these parameters, one can be found that the greater the difference, the more obvious bowing or abruption. Because this explanation is valid for the experimental findings of BiOCl_1−x_I_x_ solid solutions, so it could speculate that it also valid for BiOF_1−x_X_x_ solid solutions.

Another worth notice phenomenon is the symmetry breaking in some cases. In Fig. 4, two extreme examples are provided: the BiOCl_1−x_Br_x_ represents almost no symmetry breaking (i.e. fully obey the Vegard’s law) solid solutions; the BiOF_1−x_I_x_ represents dramatic symmetry breaking (i.e. obviously disobey the Vegard’s law) solid solutions. The similar phenomenon could be observed in BiOF_1−x_Cl_x_ and BiOF_1−x_Br_x_ solid solutions. When the content (x) of Y (Y = Cl, Br, and I) is smaller than 1/12, the symmetry breaking always exists. We think the main possible reason low content (x) of Y means impurity doping, which destroys the integrity of the interaction in the F^-^ anion plane and then make the [Bi_2_O_2_]^2+^ layer distortion, because there is no self-interaction between Y impurities. When the content (x) of Y is increasing, the self-interaction between Y anions is gradually increasing, resulting in two ordered anion (F^−^ and Y^−^) planes. On the other hand, when the content (x) of Y is larger than 11/12, the two ordered anion (F^−^ and Y^−^) planes are still maintain, owing the larger interaction of F^-^ with [Bi_2_O_2_]^2+^ layer, so the symmetry of the atomic positions is keeping. This phenomenon is more obvious if the differences between F and Y atomic properties are larger.

### Electronic and optical properties

**Figure 5:**
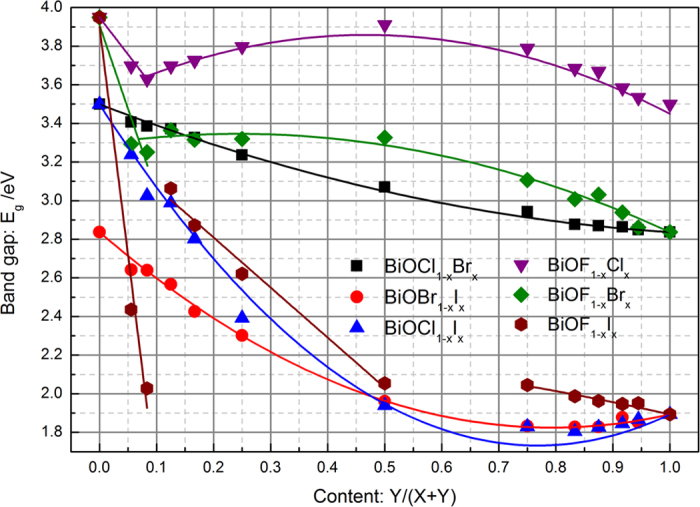
Band gap of BiOX_1__−__*x*_Y_*x*_ solid solutions as a function of content *x*.

The calculated band gaps of the pure BiOX compounds are 3.949 eV for BiOF, 3.499 eV for BiOCl, 2.837 eV for BiOBr, and 1.893 eV for BiOI, which are in good agreement with the experimental values (~3.46–3.51 eV, ~2.9 eV, and ~1.9 eV, respectively)^21^,^22^,^39^,^40^,^41^. Figure 5 shows the band gaps of the BiOX_1−*x*_Y_*x*_ solid solutions as a function of *x*. The variation trend of the band gaps calculated by the GGA + *U* method is similar to those calculated by the GGA method (Figures S7–S12 in the Supporting Information). Furthermore, the calculated results in the present work are consistent with available experimental values (Figures S13–S15)^21^,^22^,^24^,^25^,^26^,^27^,^30^,^31^,^42^. Based on the above comparison of the lattice constants and band gaps, we consider that the calculation method in the present work is reasonable and produces reliable results.

The electronic energy-band parameters of semiconductor solid solutions and their dependence on *x* are very important. However, investigation of BiOX_1−*x*_Y_*x*_-based photocatalysts has been hampered by a lack of definite knowledge about various material parameters. Therefore, it is necessary to investigate and explain the variation trends of the band gaps of BiOX_1−*x*_Y_*x*_ solid solutions. The expressions of Vegard’s law for the band gap or dielectric function constant are the same as Eq. (4) except that the symbol for the bowing parameter is *b* rather than *θ*. For the BiOCl_1−*x*_Br_*x*_ and BiOBr_1−*x*_I_*x*_ solid solutions, fitting the second-order function of Vegard’s law to the complete range of data points in Fig. 5 produces a small bowing parameter. Although the bowing parameter of the BiOCl_1−*x*_I_*x*_ solid solutions is larger, it obeys the second-order function of Vegard’s law in the complete range of *x*. For the BiOF_1−*x*_Y_*x*_ solid solutions, the band gap linearly decreases with increasing *x* for F-rich compositions, but it obeys the second-order function of Vegard’s law when *x* > 1/8 (BiOF_1−*x*_Cl_*x*_ and BiOF_1−*x*_Br_*x*_) or 3/4 (BiOF_1−*x*_I_*x*_). The variation trend of the BiOF_1−*x*_I_*x*_ solid solutions is separated into three parts: two linear variation parts and one quadratic variation part. Another interesting result is that the bowing parameters are negative for the BiOCl_1−*x*_Br_*x*_, BiOBr_1−*x*_I_*x*_, and BiOCl_1−*x*_I_*x*_ solid solutions, while the bowing parameters are positive for the BiOF_1−*x*_Y_*x*_ solid solutions. In addition, the bowing parameters decrease with increasing atomic number of Y.

**Figure 6:**
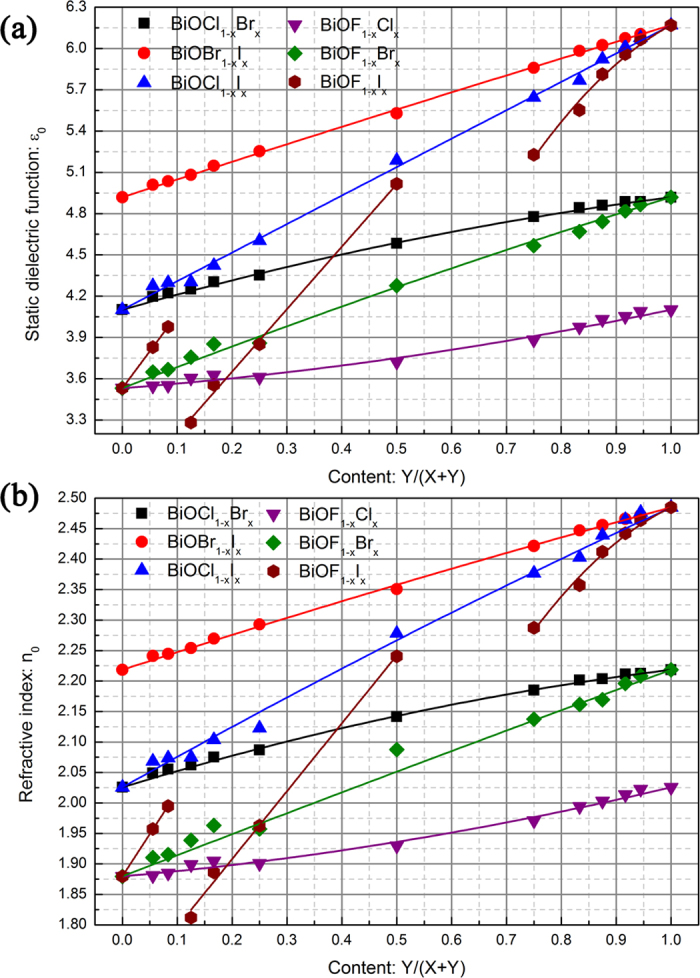
Optical properties of BiOX_1−*x*_Y_*x*_ solid solutions as a function of content *x*: (**a**) static dielectric function and (**b**) refractive index.

In addition to the variation of the band gap with *x*, the optical properties of the BiOX_1−*x*_Y_*x*_ solid solutions also show a similar variation trend. As shown in Fig. 6, the static dielectric constant (*ε*_0_) and refractive index (*n*_0_) of the BiOX_1−*x*_Y_*x*_ solid solutions increase with increasing *x*. Interestingly, expect for the BiOF_1−*x*_I_*x*_ solid solutions, the optical properties of the BiOX_1−*x*_Y_*x*_ solid solutions obey the second-order function of Vegard’s law in the complete range of *x* with very small bowing parameters. In other words, the optical properties of the BiOX_1−*x*_Y_*x*_ solid solutions (expect for the BiOF_1−*x*_I_*x*_ solid solutions) almost linearly increase with increasing *x*. For the BiOF_1−*x*_I_*x*_ solid solutions, the optical properties linearly increase with increasing *x* in the ranges 0 ≤ *x* ≤ 1/12 and 1/8 ≤ *x* ≤ 1/2, while the optical properties quadratically increase with increasing *x* in the range 3/4 ≤ *x* ≤ 1. Because optical measurements are relatively easy to perform, we fitted the calculated optical parameters as a function of composition *x*. The fitting equations are provided in Table 3.

### Possible explanation

In the present work, the properties of BiOX_1−*x*_Y_*x*_ solid solutions have an inherent connection with the differences between the physical–chemical properties of the two halogen components. The two extreme examples are BiOCl_1−*x*_Br_*x*_ and BiOF_1−*x*_I_*x*_. For the BiOCl_1−*x*_Br_*x*_ solid solutions, the miscibility temperature is very low and its properties (i.e., lattice constants, band gap, and optical properties) obey the second-order function of Vegard’s law in the complete range of *x* with small bowing parameters. In contrast, for the BiOF_1−*x*_I_*x*_ solid solutions, the miscibility temperature is very high and its properties obey different rules in different ranges of *x*: at low I content, the downward/upward bowing is so weak that there is almost linear variation, while at high I composition the bowing is stronger. Therefore, the parameters of the BiOF_1−*x*_I_*x*_ solid solutions cannot be described using a single bowing parameter, which has also been reported for other semiconductor solid solutions^43^,^44^. It is worth pointing out that BiOF_1−*x*_I_*x*_ solid solutions may show phase separation, which may change the photon-absorption mechanism.

In the above examples, Cl and Br are adjacent elements in the periodic table, while F and I are the end elements of the halogen group. In other words, the differences between Cl and Br are very slight, while the differences between F and I are very obvious. Keller *et al*. considered that Bi–X and Bi–Y bonds of different lengths coexist in a mixed crystal and the weak anion–anion interactions across the interface between two vicinal sandwiches induce the large deviation from Vegard’s law^20^, which was confirmed in the present work. We suggest that the different properties of the BiOX compounds and the variation trend differences of BiOX_1−*x*_Y_*x*_ solid solutions are determined by the physical–chemical properties of the halogens and the corresponding differences. In Table 4, we extracted the chemical and physical parameters of halogens from ref. 45 and compared their corresponding differences. Except for the differences of the electron affinity, the order of the differences of the other parameters is consistent with the order of the parameters of the BiOX_1−*x*_Y_*x*_ solid solutions, such as the slope (*a*) and the interaction parameter (Ω, or miscibility temperature) in Table 1, and the slope or the bowing parameter in Tables 2 and 3. Combining the calculated results in the present work and the data in Table 4, we conclude the following: (1) the atom radii (including van der Waals radii, covalent radii, and ionic radii) directly affect the lattice constants. Because the ratio of lattice constants *a*/*b* is mainly determined by the intralayer interaction between Bi and O atoms, it only slightly varies with *x* in all of the BiOX_1−*x*_Y_*x*_ solid solutions. However, the *c* lattice constant is mainly determined by the interlayer interactions, so its variation is related to the differences in the radii of the different halogens, and it significantly varies with *x*. (2) Both intralayer and interlayer interactions are determined by electron redistribution (i.e., electron gain or loss), which is reflected by the electronegativity or electron affinity. In the present work, we found that the order of the electron affinity difference is not consistent with the order of the parameters of the BiOX_1−*x*_Y_*x*_ solid solutions. The electron affinity indicates the ability of neutral atoms to accept electrons. In our previous work, we found that the ionic bond is stronger with increasing atomic number of the halogen in BiOX compounds^17^, indicating that BiOF exhibits an obvious mixed-bond feature. Therefore, in BiOCl_1−*x*_Br_*x*_, BiOBr_1−*x*_I_*x*_, and BiOCl_1−*x*_I_*x*_ solid solutions, the order of the electron affinity difference is consistent with the order of the solid-solution parameters. However, in BiOF_1−*x*_Y_*x*_ solid solutions, the electron affinity difference cannot be completely reflected by the variation of the solid-solution parameters. In fact, the electronegativity indicates the binding ability of a neutral atom to a valence electron, accurately reflecting the variation of the solid-solution parameters.

## Conclusions

The lattice constants, band gaps, and optical properties of BiOX_1−*x*_Y_*x*_ solid solutions have been calculated by the GGA + *U* method. The calculated lattice constants and band gaps of the BiOX_1−*x*_Y_*x*_ solid solutions agree well with the available experimental values. The calculated results show that: (1) BiOCl_1−*x*_Br_*x*_, BiOBr_1−*x*_I_*x*_, and BiOCl_1−*x*_I_*x*_ solid solutions have very small bowing parameters; thus, some of their properties almost linearly vary with *x*. (2) BiOF_1−*x*_Y_*x*_ solid solutions have very large bowing parameters. Furthermore, the properties of BiOF_1−*x*_Y_*x*_ solid solutions cannot be fitted to a single equation. In other words, its properties obey different rules in different ranges of *x*. For low Y content, the downward/upward bowing is so weak that the variation is almost linear, while at high Y content the bowing is stronger. Consequently, BiOX_1−*x*_Y_*x*_ solid solutions that do not contain fluorine are highly miscible, while those that contain fluorine are partially miscible. Therefore, BiOF_1−*x*_Y_*x*_ solid solutions have a miscibility gap or high miscibility temperature, resulting in phase separation and F/Y inhomogeneity. To provide a possible explanation, we compared and analysed the calculated results and the physical–chemical properties for varying halogen compositions, and found that the parameters of BiOX_1−*x*_Y_*x*_ solid solutions are determined by the differences of the physical–chemical properties between two halogen compositions. In this way, the large deviation from Vegard’s law in some BiOX_1−*x*_Y_*x*_ solid solutions observed in experiments can be explained. Finally, the composition ratio of BiOX_1−*x*_Y_*x*_ solid solutions can be measured or monitored using optical measurements, because their optical properties approximately linearly vary as a function of *x*, and the corresponding equations are provided. The band gap of BiOX_1−*x*_Y_*x*_ solid solutions can be tuned from 1.7 to 4.0 eV by adjusting the halogen composition, which can meet some specific requirements of BiOX-based photocatalysts. The findings in this article provide useful information for designing efficient BiOX-based photocatalysts. Summary, this article achieves the following two purposes: (1) find the underlying mechanism that BiOX_1−*x*_Y_*x*_ solid solutions obey/disobey Vegard’s law, which partially observed by different experimental researches; (2) provide some available data or formula for future experiments that want to determine or measurement the composition/band gap/optical properties of BiOX_1−*x*_Y_*x*_ solid solutions.

### Computational method and details

In the present work, all of the DFT calculations were periodic DFT calculations using the Cambridge Serial Total Energy Package (CASTEP)^46^. The interaction between core electrons (i.e., Bi: [Xe], O: [He], F: [He], Cl: [Ne], Br: [Ar], and I: [Kr]) and valence electrons was treated by the ultrasoft pseudopotential (USP) plane-wave method. The energy cutoff for the plane-wave basis wave function was 380 eV for the plane-wave basis set. The exchange–correlation interaction between valence electrons (i.e., Bi: 6 s^2^6p^3^, O: 2 s^2^2p^4^, F: 2s^2^2p^5^, Cl: 3s^2^3p^5^, Br: 4s^2^4p^5^, and I: 5s^2^5p^5^) was described by the revised Perdew–Burke–Ernzerhof functional for solids (PBEsol) with the generalized gradient approximation (GGA)^47^. To obtain accurate electronic structures, the GGA + *U* method was used to overcome the well-known shortcomings of the GGA^48^. Here, the GGA + *U* method was only used to obtain more accurate band-gap values. All of the calculations were first performed with the GGA method. The *U*_eff_ values were then determined by comparing the results of the GGA, and finally the GGA + *U* method was used to calculate the electronic and optical properties. In this way, the electronic structure characteristics obtained by the two methods are guaranteed to be as consistent as possible (except for the band-gap values). In the present work, the value of U were set as following: 4.8 eV for the p-states of Bi and O, 15 eV for the p-states of F, 7 eV for the p-states of Cl, 3.5 eV for the p-states of Br, 2.1 eV for the p-states of I. The Monkhorst–Pack scheme was used for *k*-point grid sampling in the range from 1 × 1 × 2 (for the 3 × 3 × 1 supercell) to 4 × 4 × 2 (for the pristine 1 × 1 × 1 unit cell) for the irreducible Brillouin zone. The fast Fourier transformation mesh was set in the range from 120 × 120 × 90 (for the 3 × 3 × 1 supercell) to 40 × 40 × 90 (for the pristine 1 × 1 × 1 unit cell). The minimization algorithm was the Broyden–Fletcher–Goldfarb–Shanno (BFGS) algorithm^49^. Its convergence criteria were as follows: the forces on the atoms were less than 0.03 eV/Å, the stresses on the atoms were less than 0.05 GPa, the atomic displacement was less than 1 × 10^−3^ Å, and the energy change per atom was less than 1 × 10^−5^ eV.

To construct the solid-solution model, a supercell was used. In other words, the present work used an ordered solid-solution model. Taking BiOF_17/18_Cl_1/18_ as an example, one of the F atoms was replaced by a Cl atom in the 3 × 3 × 1 BiOF supercell. For each BiOX compound, 3 × 3 × 1, 3 × 2 × 1, 2 × 2 × 1, 3 × 1 × 1, 2 × 1 × 1, and 1 × 1 × 1 supercells or cells were used to construct BiOX_1−*x*_Y_*x*_ solid solutions with different solubility or content Y/(X + Y), in which one of X atom is replaced by Y atom. In this article, X in BiOX_1−*x*_Y_*x*_ is the halogen with relatively low atomic number, while Y in BiOX_1−*x*_Y_*x*_ is the halogen with relatively high atomic number. Although disordered models constructed by the special quasirandom structures (SQS) method can produce reasonable alloying solid-solution structures, ordered models allow analysis of the microstructure and the interaction between solute atoms and solvent atoms. From a statistical point of view, the distribution of solute atoms in a solid solution is disordered. However, if the microstructure is identified, a completely disordered solid solution can only exist at high temperatures when the solute concentrations are very low. In general, although there is not a completely ordered structure in the solid solution, the local arrangement of solute atoms can be regular, which is called short-range order. Therefore, the ordered solid-solution model still has considerable significance and value in practice, and this approach was used in this study.

Another important point should be mentioned: the size of supercell and the occupying sites of Y in the supercell also impact the final total energy of models. In primary test stage of this work, we used “coarse setting” (260 eV of energy cutoff, etc. in order to save computing time) to calculate all the possible models: the size of supercell is from 1 × 1 × 1 to 3 × 3 × 1, and the possible occupation patterns are determined by the enumeration method for every supercell. After geometry optimization, we compared the total energy per cell, and chose the supercell that has the smallest total energy per cell as the candidate for the next step. For the candidate model, we used “ultra-fine setting” (380 eV of energy cutoff, etc. as mentioned above) to get the accurate result. By this way, the supercell with high symmetry and as small as possible was finally constructed for every content of Y/(X + Y).

### Supporting Information

Comparison of the calculated results with available experimental data, and comparison of the results calculated by the GGA + *U* method and the results calculated by the GGA method are available free of charge via the website of *Scientific Reports*.

## Additional Information

**How to cite this article**: Zhao, Z.-Y. *et al*. Structural, Electronic, and Optical Properties of BiOX_1−*x*_Y*_x_* (X, Y = F, Cl, Br, and I) Solid Solutions from DFT Calculations. *Sci. Rep.* **6**, 31449; doi: 10.1038/srep31449 (2016).

## Supplementary Material

Supplementary Information

## Figures and Tables

**Table 1:** Binding energies and formation enthalpies of BiOX_1−*x*_Y_*x*_ solid solutions as a function of composition.

Formula	BiOCl_1−*x*_Br_*x*_	BiOBr_1−*x*_I_*x*_	BiOCl_1−*x*_I_*x*_	BiOF_1−*x*_Cl_*x*_	BiOF_1−*x*_Br_*x*_	BiOF_1−*x*_I_*x*_
Binding Energy:*E*_*b*_(*x*) = *E*_0 _+ *ax*	*E*_0_ = −5.309*a* = 0.188	*E*_0_ = −5.120*a* = 0.199	*E*_0_ = −5.301*a* = 0.381	*E*_0_ = −5.856*a* = 0.554	*E*_0_ = −5.844*a* = 0.736	*E*_0_ = −5.828*a* = 0.931
Formation Enthalpy:Δ*H*_*f*_(*x*) = Ω*x*(1 − *x*)	Ω = 12.976*T* = 150^a^	Ω = 13.065*T* = 150^a^	Ω = 59.658*T* = 700^a^	Ω = 128.339*T* = 1490^a^	Ω = 193.265*T* = 2240^a^	Ω = 297.797*T* = 3460^a^

^a^*T* is the estimated miscibility temperature (K).

**Table 2:**
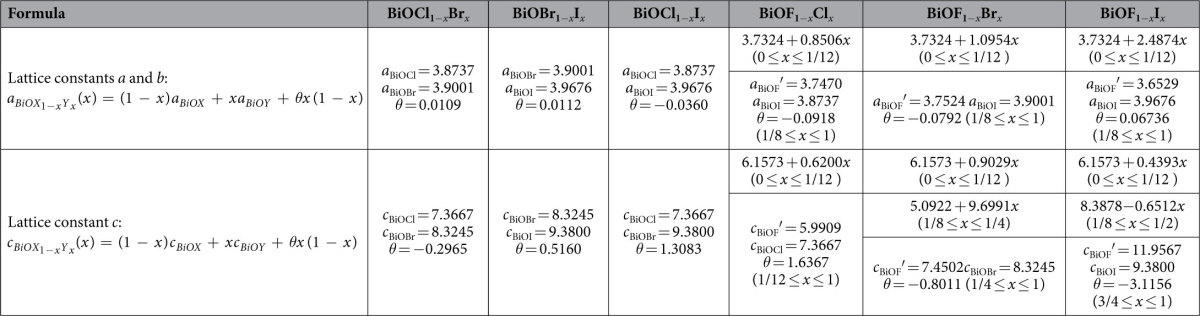
Lattice constants of BiOX_1−*x*_Y_*x*_ solid solutions as a function of composition.

**Table 3:**
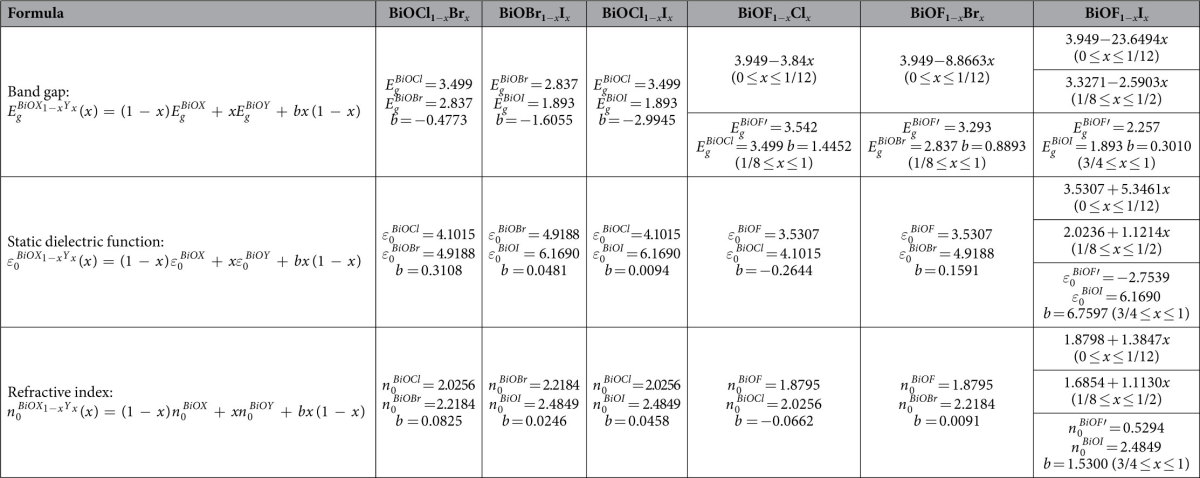
Band gaps, static dielectric functions, and refractive indices of BiOX_1−*x*_Y_*x*_ solid solutions as a function of composition.

**Table 4:** Chemical and physical parameter of halogen elements and the corresponding differences.

	van der Waals radius/Å^a^	Covalent radius/Å^a^	Ionic radius/Å^a^	Electronegativity/eV^a^	Electron affinity/eV^a^	First ionization energy/eV^a^	Mulliken electronegativity/eV^b^
F	1.47	0.60	1.33	3.98	3.4011897	17.4228	10.41199
Cl	1.75	1.00	1.81	3.16	3.612725	12.96763	8.29018
Br	1.85	1.17	1.96	2.96	3.3635882	11.8138	7.58869
I	1.98	1.36	2.20	2.66	3.0590368	10.45126	6.75515
Cl–Br	−0.10	−0.17	−0.15	0.20	0.2491368	1.15383	0.70148
Br–I	−0.13	−0.19	−0.24	0.30	0.3045514	1.36254	0.83355
Cl–I	−0.23	−0.36	−0.39	0.50	0.5536882	2.51637	1.53503
F–Cl	−0.28	−0.40	−0.48	0.82	−0.2115353	4.45517	2.12182
F–Br	−0.38	−0.57	−0.63	1.02	0.0376015	5.609	2.8233
F–I	−0.51	−0.76	−0.87	1.32	0.3421529	6.97154	3.65685

^a^Taken from ref. 45.

^b^The Mulliken electronegativity of a neutral atom is the arithmetic mean of the atomic electron affinity and the first ionization energy^50^.

## References

[b1] Briand G. G. & Burford N. Bismuth Compounds and Preparations with Biological or Medicinal Relevance. Chem. Rev. 99, 2601–2658 (1999). 11749495 10.1021/cr980425s

[b2] Kijima N. . Oxidative catalytic cracking of n-butane to lower alkenes over layered BiOCl catalyst. Appl. Catal. A 206, 237–244 (2001).

[b3] Zhang X., Ai Z., Jia F. & Zhang L. Generalized One-Pot Synthesis, Characterization, and Photocatalytic Activity of Hierarchical BiOX (X=Cl, Br, I) Nanoplate Microspheres. J. Phys. Chem. C 112, 747–753 (2008).

[b4] Deng Z., Chen D., Peng B. & Tang F. From Bulk Metal Bi to Two-Dimensional Well-Crystallized BiOX (X=Cl, Br) Micro- and Nanostructures: Synthesis and Characterization. Crystal Growth & Design 8, 2995–3003 (2008).

[b5] Zhang K. . BiOCl Sub-Microcrystals Induced by Citric Acid and Their High Photocatalytic Activities. Crystal Growth & Design 12, 793–803 (2011).

[b6] Li J., Yu Y. & Zhang L. Bismuth oxyhalide nanomaterials: layered structures meet photocatalysis. Nanoscale 6, 8473–8488 (2014). 24975748 10.1039/c4nr02553a

[b7] Cheng H., Huang B. & Dai Y. Engineering BiOX (X=Cl, Br, I) nanostructures for highly efficient photocatalytic applications. Nanoscale 6, 2009–2026 (2014). 24430623 10.1039/c3nr05529a

[b8] Ye L. . Recent advances in BiOX (X=Cl, Br and I) photocatalysts: synthesis, modification, facet effects and mechanisms. Environmental Science: Nano 1, 90–112 (2014).

[b9] Chen L. . Room-Temperature Synthesis of Flower-Like BiOX (X=Cl, Br, I) Hierarchical Structures and Their Visible-Light Photocatalytic Activity. Inorg. Chem. 52, 11118–11125 (2013). 24050663 10.1021/ic401349j

[b10] Ai Z., Ho W. & Lee S. Efficient Visible Light Photocatalytic Removal of NO with BiOBr-Graphene Nanocomposites. J. Phys. Chem. C 115, 25330–25337 (2011).

[b11] Zhang X., Zhang L. & Xie T., Wang, D. Low-Temperature Synthesis and High Visible-Light-Induced Photocatalytic Activity of BiOI/TiO_2_ Heterostructures. J. Phys. Chem. C 113, 7371–7378 (2009).

[b12] Zhang X. & Zhang L. Electronic and Band Structure Tuning of Ternary Semiconductor Photocatalysts by Self Doping: The Case of BiOI. J. Phys. Chem. C 114, 18198–18206 (2010).

[b13] Shenawi-Khalil S. . A Novel Heterojunction BiOBr/Bismuth Oxyhydrate Photocatalyst with Highly Enhanced Visible Light Photocatalytic Properties. J. Phys. Chem. C 116, 11004–11012 (2012).

[b14] Chen J. . The dominant {001} facet-dependent enhanced visible-light photoactivity of ultrathin BiOBr nanosheets. Phys. Chem. Chem. Phys. 16, 20909–20914 (2014). 25171684 10.1039/c4cp02972k

[b15] Yue D. . Enhancement of visible photocatalytic performances of a Bi_2_MoO_6_-BiOCl nanocomposite with plate-on-plate heterojunction structure. Phys. Chem. Chem. Phys. 16, 26314–26321 (2014). 25367447 10.1039/c4cp03865g

[b16] Zhang X. . The stabilities and electronic structures of single-layer bismuth oxyhalides for photocatalytic water splitting. Phys. Chem. Chem. Phys. 16, 25854–25861 (2014). 25354143 10.1039/c4cp03166k

[b17] Zhao Z.-Y. & Dai W.-W. Structural, Electronic, and Optical Properties of Eu-Doped BiOX (X=F, Cl, Br, I): A DFT + U Study. Inorg. Chem. 53, 13001–13011 (2014). 25413500 10.1021/ic5021059

[b18] Maeda K. . Overall Water Splitting on (Ga_1−x_Zn_x_)(N_1−x_O_x_) Solid Solution Photocatalyst: Relationship between Physical Properties and Photocatalytic Activity. J. Phys. Chem. B 109, 20504–20510 (2005). 16853653 10.1021/jp053499y

[b19] Maeda K. . Photocatalytic activity of (Ga_1−x_Zn_x_)(N_1−x_O_x_) for visible-light-driven H_2_ and O_2_ evolution in the presence of sacrificial reagents. J. Phys. Chem. C 112, 3447–3452 (2008).

[b20] Keller E. & Krämer V. A Strong Deviation from Vegard’s Rule: X-Ray Powder Investigations of the Three Quasi-Binary Phase Systems BiOX-BiOY (X, Y=Cl, Br, I) Zeitschrift für Naturforschung B 60b, 1255 (2005).

[b21] Wang W., Huang F. & Lin X. xBiOI–(1−x)BiOCl as efficient visible-light-driven photocatalysts. Scripta Mater. 56, 669–672 (2007).

[b22] Wang W., Huang F., Lin X. & Yang J. Visible-light-responsive photocatalysts xBiOBr–(1−x)BiOI. Catal. Commun. 9, 8–12 (2008).

[b23] Huang K. J., Liu H. G. & Xie C. S. Synthesis, Characterization and Visible Light Photocatalytic Properties of BiOCl_0.2_Br_0.1_I_0.7_. In: Advances in Mechanical Engineering, Pts 1–3 (eds Zhou M.). Trans Tech Publications Ltd (2011).

[b24] Jia Z., Wang F., Xin F. & Zhang B. Simple Solvothermal Routes to Synthesize 3D BiOBr_x_I_1−x_ Microspheres and Their Visible-Light-Induced Photocatalytic Properties. Ind. Eng. Chem. Res. 50, 6688–6694 (2011).

[b25] Liu Y. . Composition Dependence of the Photocatalytic Activities of BiOCl_1−x_Br_x_ Solid Solutions under Visible Light. Chem. Eur. J. 17, 9342–9349 (2011). 21732448 10.1002/chem.201100952

[b26] Shenawi-Khalil S. . A new family of BiO(Cl_x_Br_1−x_) visible light sensitive photocatalysts. Catal. Commun. 12, 1136–1141 (2011).

[b27] Dong F. . Room temperature synthesis and highly enhanced visible light photocatalytic activity of porous BiOI/BiOCl composites nanoplates microflowers. J. Hazard. Mater. 219–220, 26–34 (2012). 10.1016/j.jhazmat.2012.03.015 22502896

[b28] Gnayem H. & Sasson Y. Hierarchical Nanostructured 3D Flowerlike BiOCl_x_Br_1−x_ Semiconductors with Exceptional Visible Light Photocatalytic Activity. ACS Catal. 3, 186–191 (2013).

[b29] Lei Y., Wang G., Guo P. & Song H. The Ag–BiOBrxI1−x composite photocatalyst: Preparation, characterization and their novel pollutants removal property. Appl. Surf. Sci. 279, 374–379 (2013).

[b30] Mao X.-m. & Fan C.-m. Effect of light response on the photocatalytic activity of BiOCl_x_Br_1−x_ in the removal of Rhodamine B from water. International Journal of Minerals, Metallurgy, and Materials 20, 1089–1096 (2013).

[b31] Ren K. . Synthesis of the bismuth oxyhalide solid solutions with tunable band gap and photocatalytic activities. Dalton Trans. 42, 9706–9712 (2013). 23680910 10.1039/c3dt50498k

[b32] Zhang B. . Efficient adsorption and photocatalytic pceerformance of flower-like three-dimensional (3D) I-doped BiOClBr photocatalyst. Catal. Commun. 36, 25–30 (2013).

[b33] Huang S.-T. . Synthesis, characterization, photocatalytic activity of visible-light-responsive photocatalysts BiOxCly/BiOmBrn by controlled hydrothermal method. J. Mol. Catal. A: Chem. 391, 105–120 (2014).

[b34] Liu H. . Graphene sheets grafted three-dimensional BiOBr_0.2_I_0.8_ microspheres with excellent photocatalytic activity under visible light. J. Hazard. Mater. 266, 75–83 (2014). 24374567 10.1016/j.jhazmat.2013.12.013

[b35] Zhang H., Liu L. & Zhou Z. Towards better photocatalysts: first-principles studies of the alloying effects on the photocatalytic activities of bismuth oxyhalides under visible light. Phys. Chem. Chem. Phys. 14, 1286–1292 (2012). 22146949 10.1039/c1cp23516h

[b36] Chen S. . Compositional dependence of structural and electronic properties of Cu_2_ZnSn(S, Se)_4_ alloys for thin film solar cells. Phys. Rev. B 83, 125201 (2011).

[b37] Shu Q. . Cu_2_Zn(Sn, Ge)Se_4_ and Cu_2_Zn(Sn, Si)Se_4_ alloys as photovoltaic materials: Structural and electronic properties. Phys. Rev. B 87, 115208 (2013).

[b38] Vegard L. Die Konstitution der Mischkristalle und die Raumfüllung der Atome. Z. Phys. A 5, 17–26 (1921).

[b39] Zhang K.-L. . Study of the electronic structure and photocatalytic activity of the BiOCl photocatalyst. Appl. Catal. B 68, 125–129 (2006).

[b40] Henle J. . Nanosized BiOX (X=Cl, Br, I) Particles Synthesized in Reverse Microemulsions. Chem. Mater. 19, 366–373 (2007).

[b41] Lin X. . Photocatalytic activity of a novel Bi-based oxychloride catalyst Na_0.5_Bi_1.5_O_2_Cl. Solid State Sci. 9, 944–949 (2007).

[b42] Li T. B. . New photocatalyst BiOCl/BiOI composites with highly enhanced visible light photocatalytic performances. Dalton Trans. 40, 6751–6758 (2011). 21617792 10.1039/c1dt10471c

[b43] Moses P. G. & Van de Walle C. G. Band bowing and band alignment in InGaN alloys. Appl. Phys. Lett. 96, 021908–021903 (2010).

[b44] McCluskey M. D. . Large band gap bowing of In_x_Ga_1−x_N alloys. Appl. Phys. Lett. 72, 2725–2726 (1998).

[b45] Haynes W. M. CRC Handbook of Chemistry and Physics. 95th edition. CRC Press, (2014).

[b46] Clark S. J. . First principles methods using CASTEP. Z. Kristallogr. 220, 567–570 (2005).

[b47] Perdew J. P. . Restoring the Density-Gradient Expansion for Exchange in Solids and Surfaces. Phys. Rev. Lett. 100, 136406 (2008). 18517979 10.1103/PhysRevLett.100.136406

[b48] Anisimov V. I., Zaanen J. & Andersen O. K. Band theory and Mott insulators: Hubbard U instead of Stoner I. Phys. Rev. B 44, 943 (1991). 10.1103/physrevb.44.943 9999600

[b49] Pfrommer B. G., Câté M., Louie S. G. & Cohen M. L. Relaxation of Crystals with the Quasi-Newton Method. J. Comput. Phys. 131, 233–240 (1997).

[b50] Butler M. A. & Ginley D. S. Prediction of Flatband Potentials at Semiconductor‐Electrolyte Interfaces from Atomic Electronegativities. J. Electrochem. Soc. 125, 228–232 (1978).

