# Supplementary material for: Structural, Electronic, and Optical Properties of BiOX1−xYx (X, Y = F, Cl, Br, and I) Solid Solutions from DFT Calculations
Source: Sci Rep. 2016 Aug 23;6:31449. doi: 10.1038/srep31449 (PMC4994000; doi:10.1038/srep31449)
Supplement: Supplementary Information [file srep31449-s1.pdf]

Supporting Information

for

Structural, Electronic, and Optical Properties of $BiOX_{1-x}Y_x$ (X, Y = F, Cl, Br, and I) Solid Solutions from DFT Calculations

Zong-Yan Zhao[1,3,*], Qing-Lu Liu[2], Wen-Wu Dai[3]

[1] Yunnan Key Laboratory of Micro/Nano Materials & Technology, School of Materials Science and Engineering, Yunnan Univeristy, Kunming 650504, People's Republic of China

[2] Key Laboratory of Nanodevices and Applications, Suzhou Institute of Nano-Tech and Nano-Bionics, Chinese Academy of Sciences, Suzhou 215123, People's Republic of China

[3] Faculty of Materials Science and Engineering, Kunming University of Science and Technology, Kunming 650093, People's Republic of China

[*]Corresponding author: Tel.: +86-871-65919924. E-mail address: zzy@kmust.edu.cn

Table R1. Comparison of lattice parameters, binding energy, and band gap of BiOX (X = F, Cl, Br, and I) calculated by two pseudopotentials

		BiOF	BiOCl	BiOBr	BiOI
Lattice parameter /Å	Ultrasoft pseudopotentials	a=b=3.7347 c=6.1573	a=b=3.8737 c=7.3667	a=b=3.9001 c=8.3245	a=b=3.9676 c=9.3800
	On the fly pseudopotentials	a=b=3.7347 c=6.1573	a=b=3.8983 c=7.3444	a=b=3.9204 c=8.3499	a=b=3.9880 c=9.3936
	Experimental measurement	a=b= 3.7469 c= 6.226	a=b= 3.892 c= 7.375	a=b= 3.927 c= 8.106	a=b= 3.9952 c= 9.1515
Binding energy /eV.cell^{-1}	Ultrasoft pseudopotentials	24.4008	21.7360	21.0396	20.1035
	On the fly pseudopotentials	22.7741	20.3073	19.6458	18.8652
Band gap /eV	Ultrasoft pseudopotentials	3.949	3.499	2.837	1.893
	On the fly pseudopotentials	3.788	3.431	2.810	1.806
	Experimental measurement	~4.0	~3.5	~2.8	~1.9

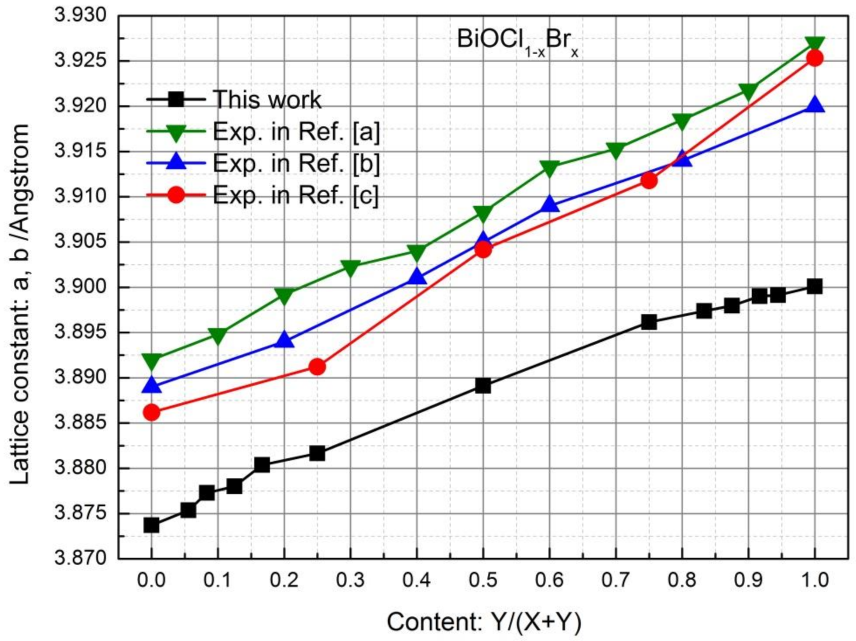

Figure S1. Comparison of lattice constant a/b of $BiOCl_{1-x}Br_x$ solid solution in the present work with experimental measurements in References (a: [1], b: [2], c: [3])

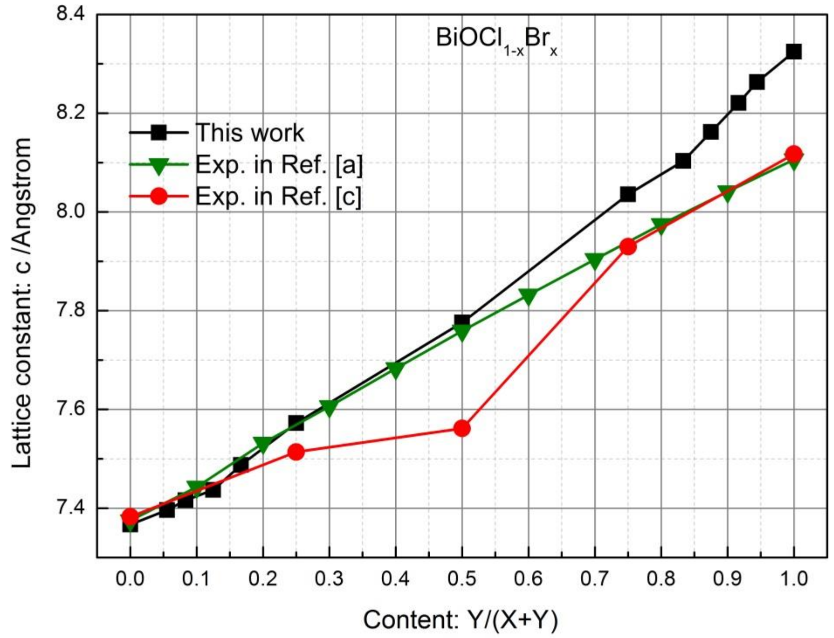

Figure S2. Comparison of lattice constant c of $BiOCl_{1-x}Br_x$ solid solution in the present work with experimental measurements in References (a: [1], c: [3])

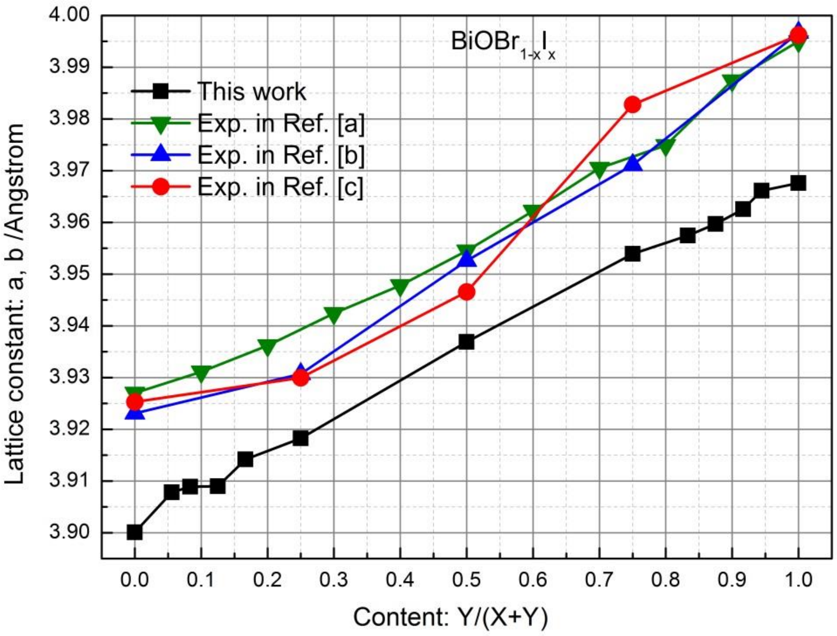

Figure S3. Comparison of lattice constant a/b of $BiOBr_{1-x}I_x$ solid solution in the present work with experimental measurements in References (a: [1], b: [4], c: [3])

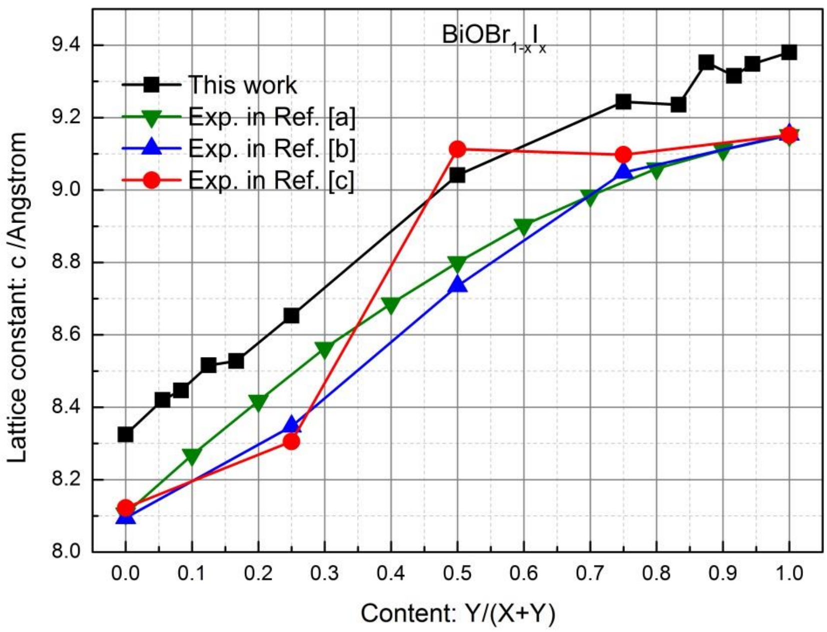

Figure S4. Comparison of lattice constant c of $BiOBr_{1-x}I_x$ solid solution in the present work with experimental measurements in References (a: [1], b: [4], c: [3])

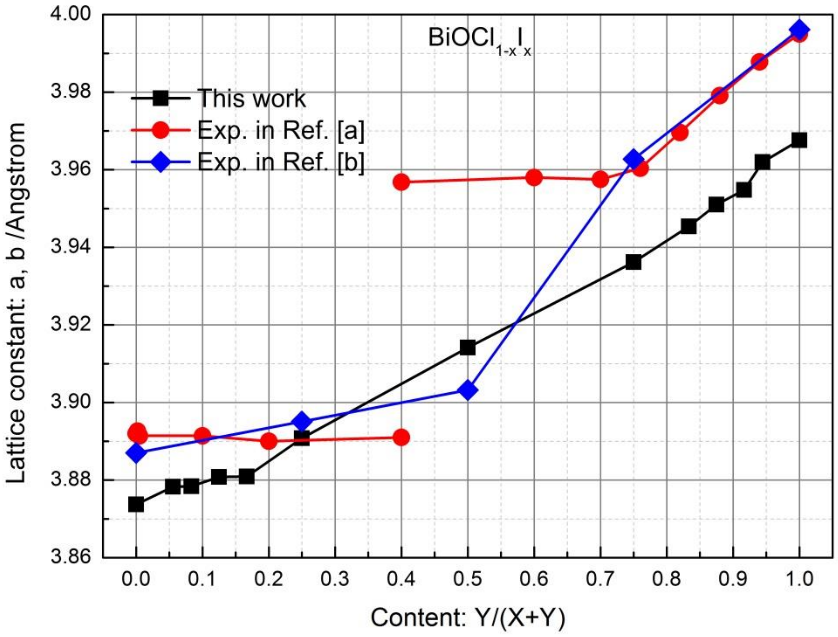

Figure S5. Comparison of lattice constant a/b of $BiOCl_{1-x}I_x$ solid solution in the present work with experimental measurements in References (a: [1], b: [3])

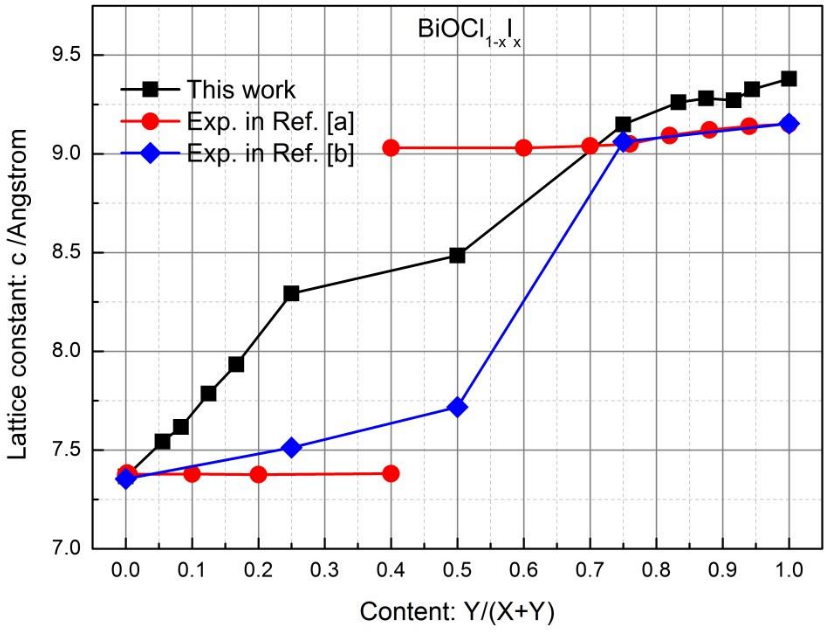

Figure S6. Comparison of lattice constant c of $BiOCl_{1-x}I_x$ solid solution in the present work with experimental measurements in References (a: [1], b: [3])

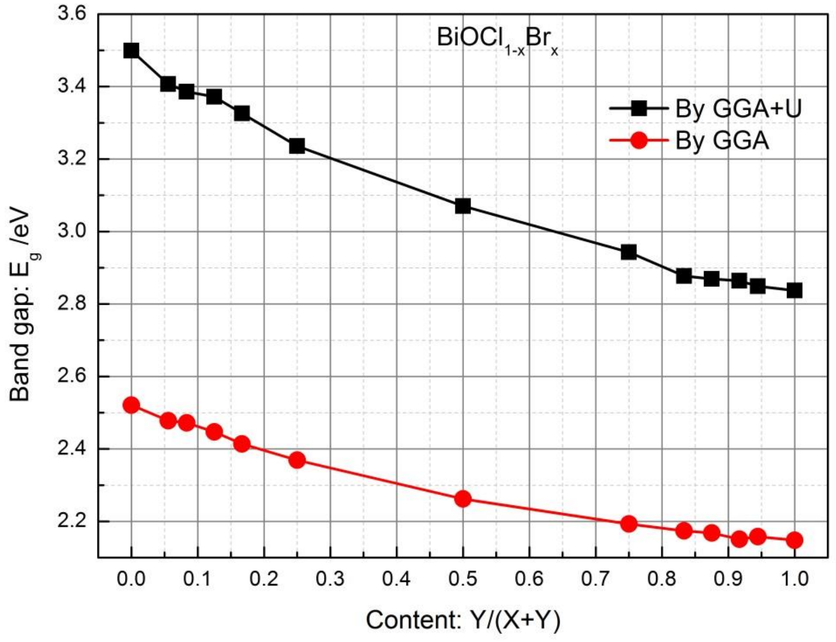

Figure S7. Comparison of band gaps of $BiOCl_{1-x}Br_x$ solid solutions by GGA+U method and GGA method

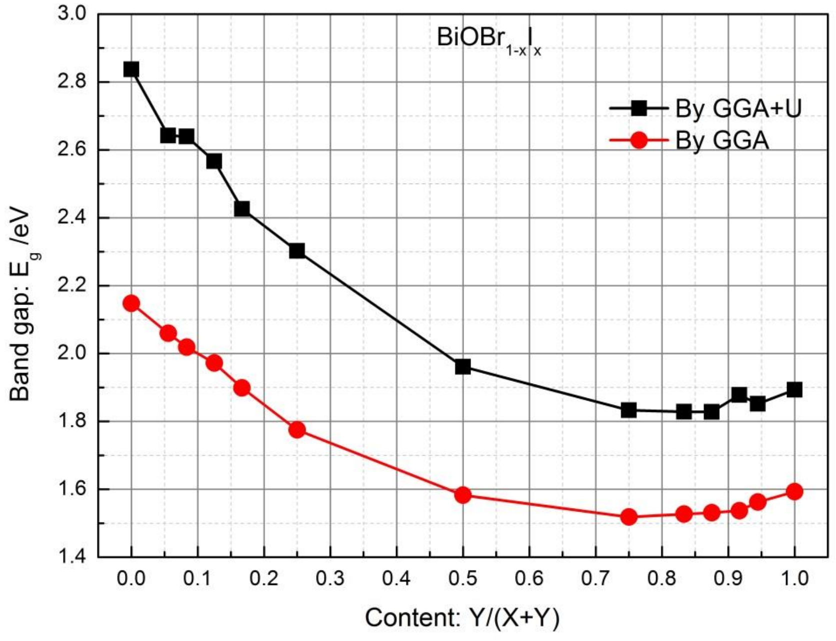

Figure S8. Comparison of band gaps of $BiOBr_{1-x}I_x$ solid solutions by GGA+U method and GGA method

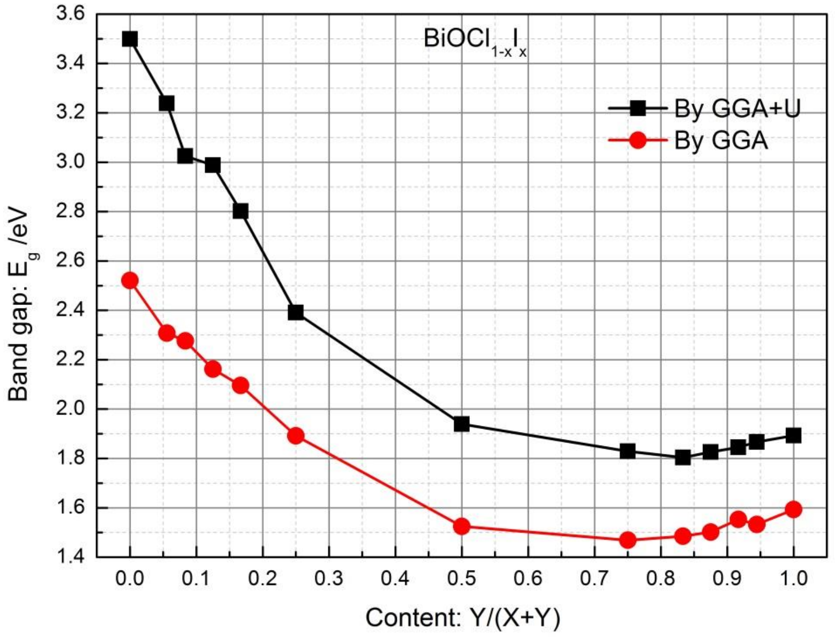

Figure S9. Comparison of band gaps of $BiOCl_{1-x}I_x$ solid solutions by GGA+U method and GGA method

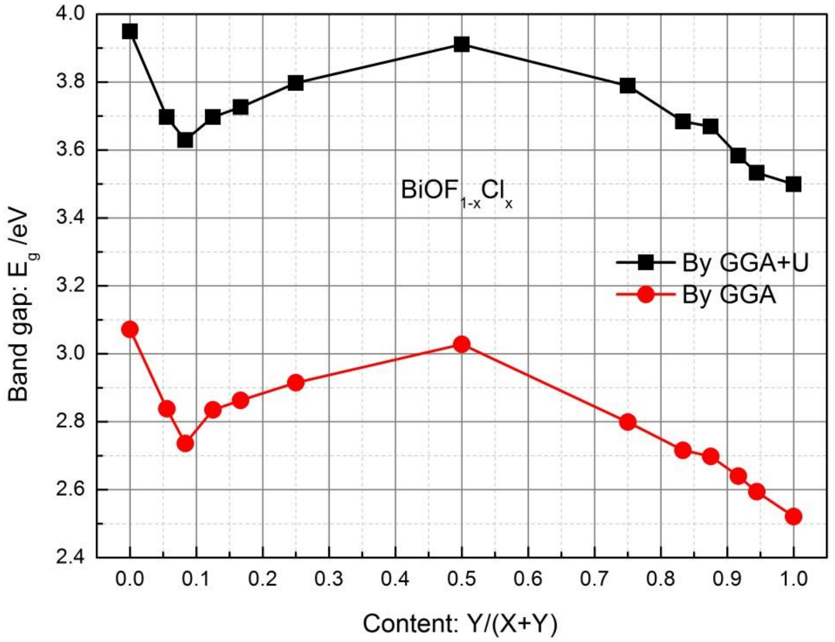

Figure S10. Comparison of band gaps of $BiOF_{1-x}Cl_x$ solid solutions by GGA+U method and GGA method

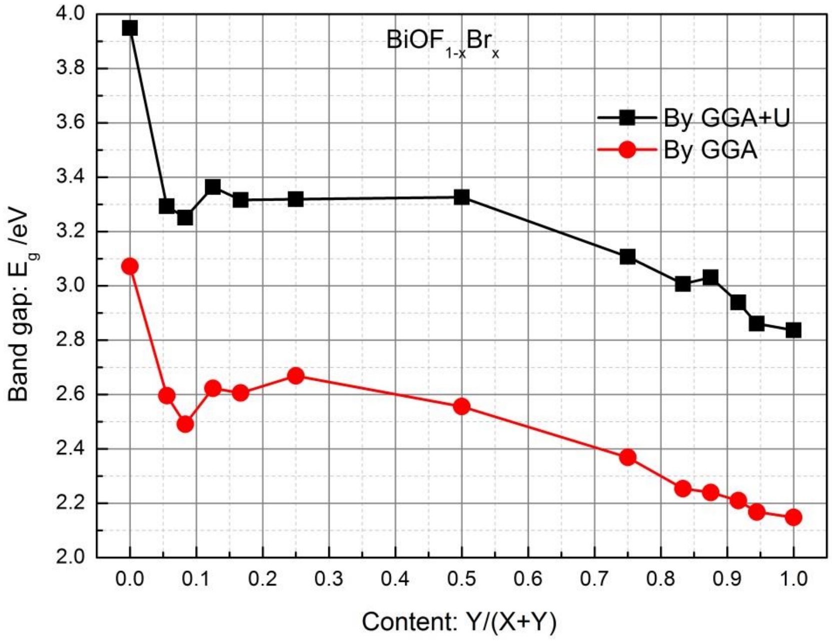

Figure S11. Comparison of band gaps of $BiOF_{1-x}Br_x$ solid solutions by GGA+U method and GGA method

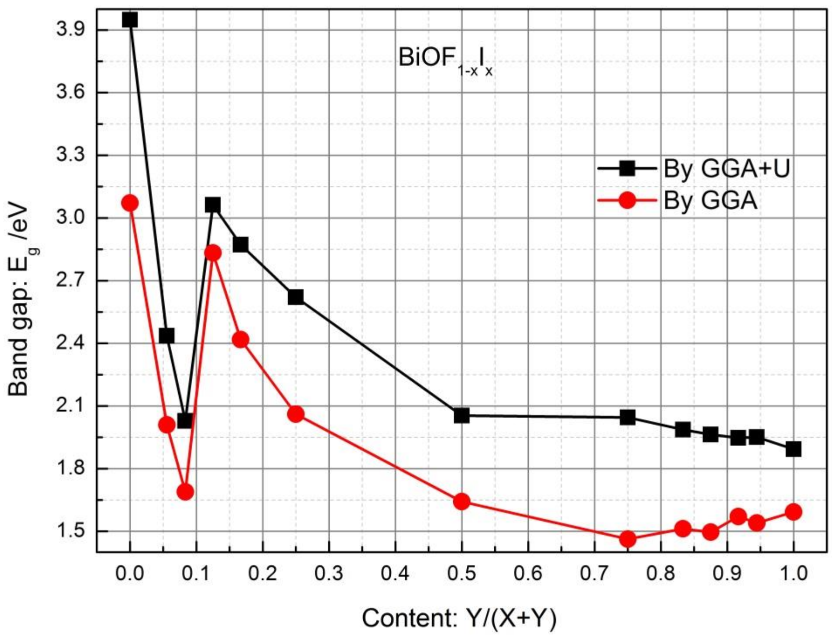

Figure S12. Comparison of band gaps of $BiOF_{1-x}I_x$ solid solutions by GGA+U method and GGA method

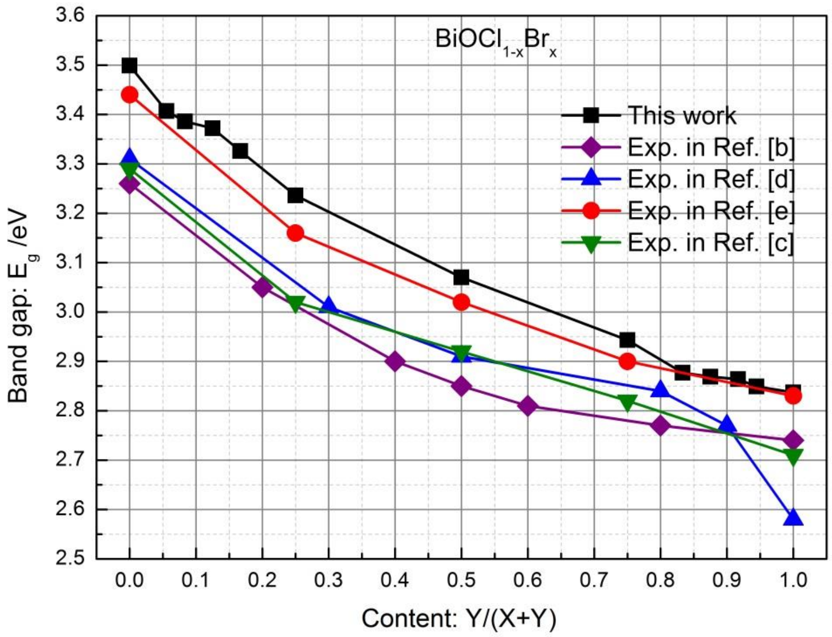

Figure S13. Comparison of band gap of $BiOCl_{1-x}Br_x$ solid solution in the present work with experimental measurements in References (b: [2], c: [3], d: [5], e: [6])

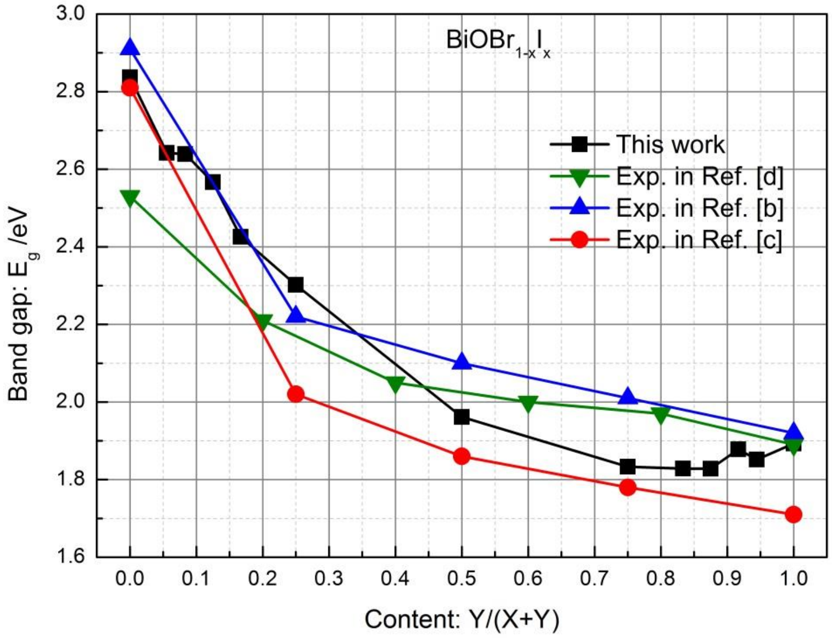

Figure S14. Comparison of band gap of $BiOBr_{1-x}I_x$ solid solution in the present work with experimental measurements in References (b: [4], c: [3], d: [7])

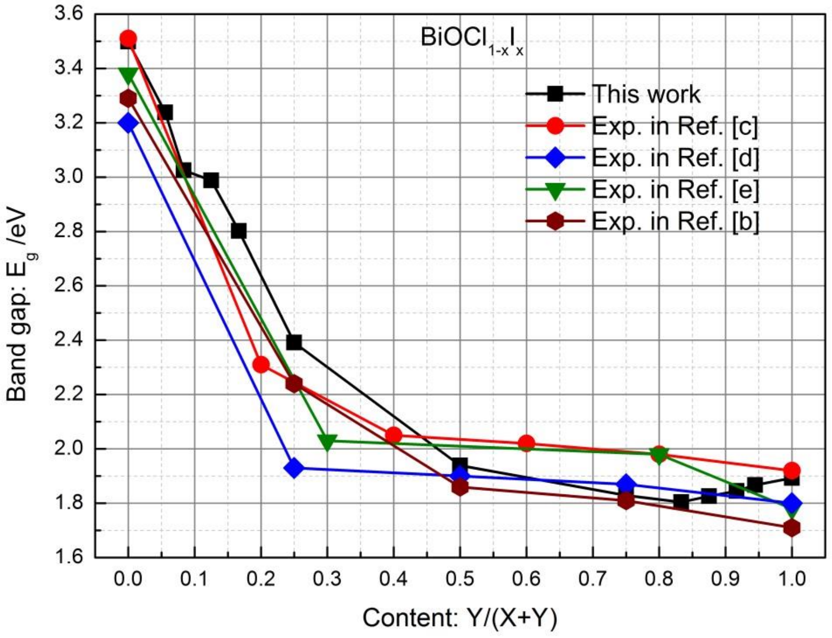

Figure S15. Comparison of band gap of $BiOCl_{1-x}Br_x$ solid solution in the present work with experimental measurements in References (b: [3], c: [8], d: [9], e: [10])

REFERENCES

(1) Keller, E.; Krämer, V., A Strong Deviation from Vegard's Rule: X-Ray Powder Investigations of the Three Quasi-Binary Phase Systems BiOX-BiOY (X, Y = Cl, Br, I) *Zeitschrift für Naturforschung B* **2005**, *60b*, 1255.

(2) Liu, Y.; Son, W.-J.; Lu, J.; Huang, B.; Dai, Y.; Whangbo, M.-H., Composition Dependence of the Photocatalytic Activities of $BiOCl_{1-x}Br_x$ Solid Solutions under Visible Light. *Chem. Eur. J.* **2011**, *17*, 9342-9349.

(3) Ren, K.; Liu, J.; Liang, J.; Zhang, K.; Zheng, X.; Luo, H.; Huang, Y.; Liu, P.; Yu, X., Synthesis of the Bismuth Oxyhalide Solid Solutions with Tunable Band Gap and Photocatalytic Activities. *Dalton Trans.* **2013**, *42*, 9706-9712.

(4) Wang, W.; Huang, F.; Lin, X.; Yang, J., Visible-Light-Responsive Photocatalysts xBiOBr–(1−x)BiOI. *Catal. Commun.* **2008**, *9*, 8-12.

(5) Shenawi-Khalil, S.; Uvarov, V.; Kritsman, Y.; Menes, E.; Popov, I.; Sasson, Y., A New Family of $BiO(Cl_xBr_{1-x})$ Visible Light Sensitive Photocatalysts. *Catal. Commun.* **2011**, *12*, 1136-1141.

(6) Mao, X.-m.; Fan, C.-m., Effect of Light Response on the Photocatalytic Activity of $BiOCl_xBr_{1-x}$in the Removal of Rhodamine B from Water. *International Journal of Minerals, Metallurgy, and Materials* **2013**, *20*, 1089-1096.

(7) Jia, Z.; Wang, F.; Xin, F.; Zhang, B., Simple Solvothermal Routes to Synthesize 3D $BiOBr_xI_{1-x}$ Microspheres and Their Visible-Light-Induced Photocatalytic Properties. *Ind. Eng. Chem. Res.* **2011**, *50*, 6688-6694.

(8) Wang, W.; Huang, F.; Lin, X., xBiOI–(1-x)BiOCl as Efficient Visible-Light-Driven Photocatalysts. *Scripta Mater.* **2007**, *56*, 669-672.

(9) Dong, F.; Sun, Y.; Fu, M.; Wu, Z.; Lee, S. C., Room Temperature Synthesis and Highly Enhanced Visible Light Photocatalytic Activity of Porous BiOI/BiOCl Composites Nanoplates Microflowers. *J. Hazard. Mater.* **2012**, *219–220*, 26-34.

(10) Li, T. B.; Chen, G.; Zhou, C.; Shen, Z. Y.; Jin, R. C.; Sun, J. X., New Photocatalyst BiOCl/BiOI Composites with Highly Enhanced Visible Light Photocatalytic Performances. *Dalton Trans.* **2011**, *40*, 6751-6758.
